# Scaffold Hybridization Strategy Leads to the Discovery of Dopamine D_3_ Receptor-Selective or Multitarget Bitopic Ligands Potentially Useful for Central Nervous System Disorders

**DOI:** 10.1021/acschemneuro.1c00368

**Published:** 2021-09-16

**Authors:** Alessandro Bonifazi, Amy H. Newman, Thomas M. Keck, Silvia Gervasoni, Giulio Vistoli, Fabio Del Bello, Gianfabio Giorgioni, Pegi Pavletić, Wilma Quaglia, Alessandro Piergentili

**Affiliations:** † Medicinal Chemistry Section, Molecular Targets and Medications Discovery Branch, National Institute on Drug Abuse—Intramural Research Program, National Institutes of Health, 333 Cassell Drive, Baltimore, Maryland 21224, United States; ‡ Department of Chemistry & Biochemistry, Department of Molecular & Cellular Biosciences, Rowan University, 201 Mullica Hill Rd, Glassboro, New Jersey 08028, United States; § School of Pharmacy, Medicinal Chemistry Unit, University of Camerino, Via S. Agostino 1, Camerino 62032, Italy; ∥ Department of Pharmaceutical Sciences, University of Milan, Via Mangiagalli 25, Milano 20133, Italy

**Keywords:** dopamine D_3_ receptors, bitopic ligands, multitarget compounds, central nervous system disorders, docking studies

## Abstract

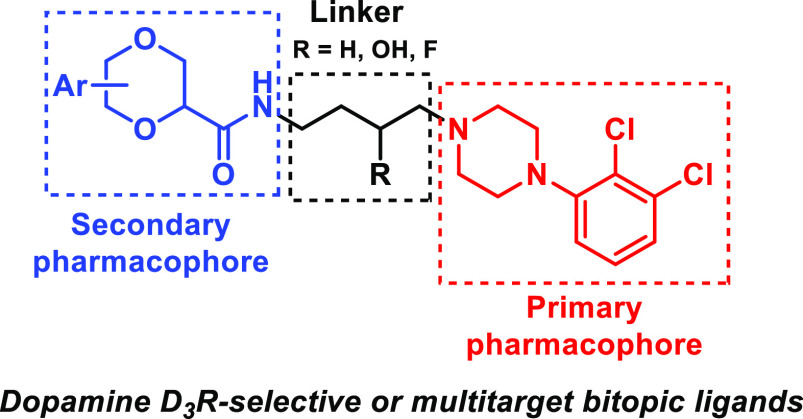

In the search for novel bitopic compounds targeting the dopamine D_3_ receptor (D_3_R), the *N*-(2,3-dichlorophenyl)piperazine nucleus (primary pharmacophore) has been linked to the 6,6- or 5,5-diphenyl-1,4-dioxane-2-carboxamide or the 1,4-benzodioxane-2-carboxamide scaffold (secondary pharmacophore) by an unsubstituted or 3-F-/3-OH-substituted butyl chain. This scaffold hybridization strategy led to the discovery of potent D_3_R-selective or multitarget ligands potentially useful for central nervous system disorders. In particular, the 6,6-diphenyl-1,4-dioxane derivative **3** showed a D_3_R-preferential profile, while an interesting multitarget behavior has been highlighted for the 5,5-diphenyl-1,4-dioxane and 1,4-benzodioxane derivatives **6** and **9**, respectively, which displayed potent D_2_R antagonism, 5-HT_1A_R and D_4_R agonism, as well as potent D_3_R partial agonism. They also behaved as low-potency 5-HT_2A_R antagonists and 5-HT_2C_R partial agonists. Such a profile might be a promising starting point for the discovery of novel antipsychotic agents.

## Introduction

Dopamine, a widely distributed neurotransmitter in both vertebrates and invertebrates, is a catecholamine produced in several areas of the brain. It plays several physiological roles by interacting with specific receptors, which are members of the G protein-coupled receptor (GPCR) superfamily and are classified into two main groups, namely, D_1_-like, including D_1_ and D_5_ receptors (D_1_Rs and D_5_Rs), and D_2_-like, comprising D_2_, D_3_, and D_4_ receptors (D_2_Rs, D_3_Rs, and D_4_Rs).^1^ There is a high degree of amino acid homology within the binding sites of the D_2_-like receptors, especially between the D_2_R and D_3_R subtypes.^2^ Hence, the discovery of novel D_3_R-selective compounds is still a challenge and, to date, no highly selective D_3_R agonists or antagonists are available for therapeutic use in humans.^3^ Moreover, these receptors share high homology with other receptor systems, including serotonergic receptors.^4^

The discovery of D_3_R-preferential compounds, the cloning of D_3_R gene, and a better understanding of D_3_R biology have made clear that D_3_R pharmacology is deeply different from that of the other D_2_-like receptor subtypes.^5−7^ The resolved crystal structure of D_3_R in complex with the D_2_R/D_3_R-specific antagonist eticlopride has provided greater clarity on the molecular basis of ligand–receptor interactions and played an important role in the design of novel D_3_R-selective ligands.^8,9^

Altered D_3_R signaling is associated with a number of pathological conditions, including substance use disorder, Parkinson’s disease (PD), schizophrenia, depression, and restless leg syndrome.^10−17^ Each of these conditions may benefit from pharmacological manipulation of D_3_R signaling, and selective D_3_R ligands may represent important pharmacological tools that might provide further information toward the D_3_R (patho)physiological role and that lack motor side effects associated with D_2_R blockade.^17−20^

**Figure 1:**
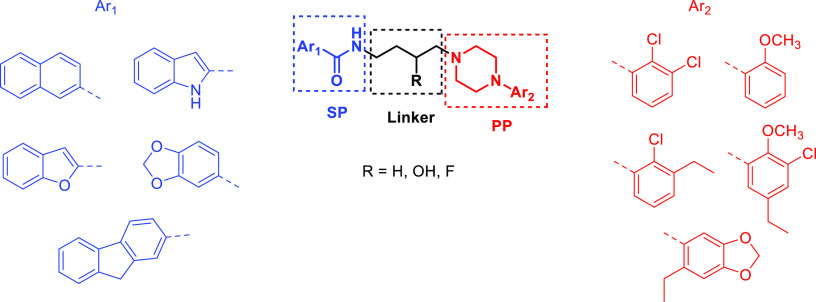
General structure of selective D_3_R bitopic ligands^10,24^ and representative PPs (Ar_2_ in red), SPs (Ar_1_ in blue), and linkers.

A promising strategy for improving D_3_R selectivity involves the development of bitopic ligands that bear an aryl piperazine as the primary pharmacophore (PP) linked, via an alkyl chain of specified length and composition, to an arylcarboxamide as the secondary pharmacophore (SP) and has led to the discovery of potent and selective D_3_R agents (Figure 1).^21−24^

Molecular modeling studies based on the D_3_R crystal structure demonstrated that the PP recognizes the orthosteric binding site of the receptor, nicely overlapping with eticlopride, whereas the SP binds to the less conserved secondary binding pocket. Moreover, the unsubstituted or (3-OH or 3-F) substituted butyl linkers can favor (or disfavor) the correct binding pose of the ligand.^24^

D_2_-like multitarget ligands have also demonstrated their potential as therapeutically useful agents,^25^ especially in PD and schizophrenia treatment.^26,27^ In particular, combining D_2_R/D_3_R antagonism, 5-HT_1A_ receptor (5-HT_1A_R) agonism, and 5-HT_2A_R antagonism proved to be favorable in the management of schizophrenia.^5,28^ Moreover, the simultaneous D_2_-like receptor and 5-HT_1A_R activation might be advantageous in PD therapy. In this case, the 5-HT_1A_R stimulation might reduce the dyskinetic side effects induced by D_2_-like receptor activation.^29,30^

**Figure 2:**
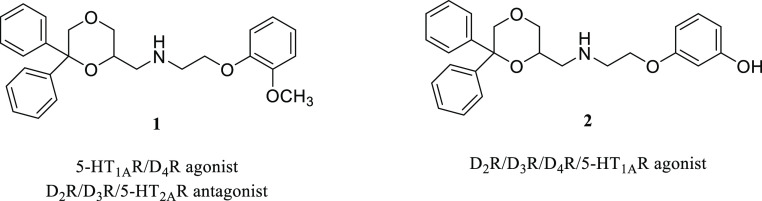
Chemical structures of the multitarget 1,4-dioxane derivatives **1** and **2**. Adapted from ref (41). Copyright 2019 ACS Chemical Neuroscience.

Over the last decade, we have demonstrated that the 1,4-dioxane nucleus represents a bioversatile carrier of ligands interacting with different receptor systems,^31−40^ including D_2_-like receptors.^41^ In particular, two properly substituted 1,4-dioxane compounds endowed with the fruitful multitarget combination of 5-HT_1A_R/D_4_R agonism and D_2_R/D_3_R/5-HT_2A_R antagonism (compound **1**) or D_2_R/D_3_R/D_4_R/5-HT_1A_R agonism (compound **2**) have been discovered as potential starting points to develop new pharmacological tools for schizophrenia and PD therapy, respectively^41^ (Figure 2).

**Figure 3:**
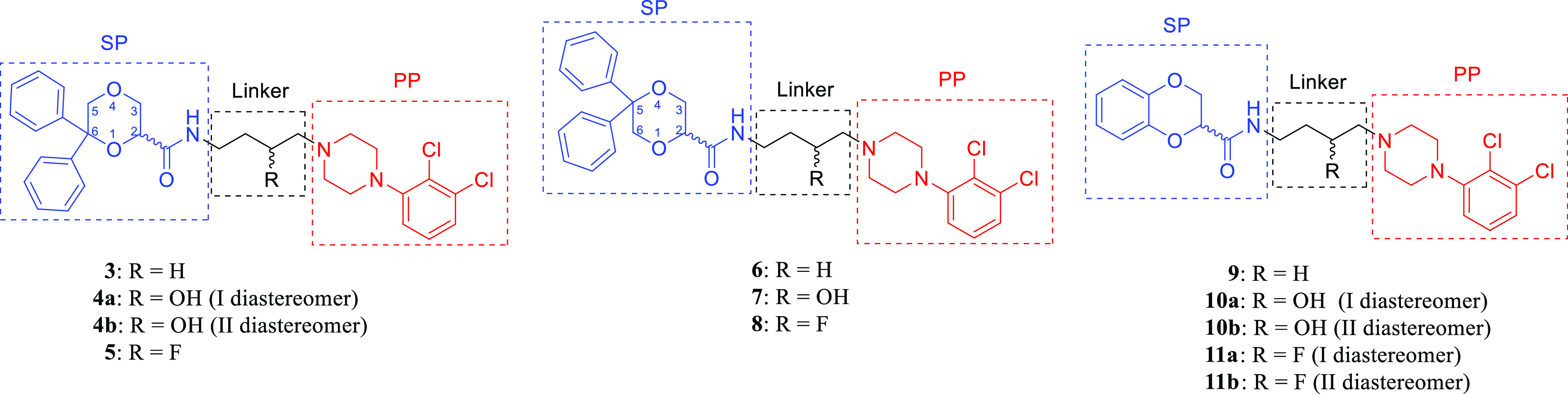
Chemical structures of compounds **3–11** (SP = secondary pharmacophore, PP = primary pharmacophore).

Based on these observations, the aim of the present investigation was to evaluate the utility of the substituted 1,4-dioxane scaffold as a SP of bitopic compounds targeting D_3_R, in order to obtain new D_3_R-selective or multitarget agents. Therefore, hybrid ligands bearing the *N*-(2,3-dichlorophenyl)piperazine nucleus, one of the most common PPs present in selective D_3_R partial agonists and antagonists,^24^ linked by an unsubstituted or 3-F-/3-OH-substituted butyl chain, to the 6,6- or 5,5-diphenyl-1,4-dioxane-2-carboxamide (compounds **3–8**) or the 1,4-benzodioxane-2-carboxamide (compounds **9–11**) scaffold as the SP, have been synthesized (Figure 3). All compounds were tested at human D_2_-like receptor subtypes in radioligand competition binding assays. Moreover, the biological profiles of the most promising compounds **3**, **6**, and **9** were further evaluated in binding assays at other selected targets (D_1_R, 5-HT_1A_R, 5-HT_2A_R, and 5-HT_2C_R) and in functional assays at receptors in which they showed the highest affinities.

## Results and Discussion

**Scheme 1:**
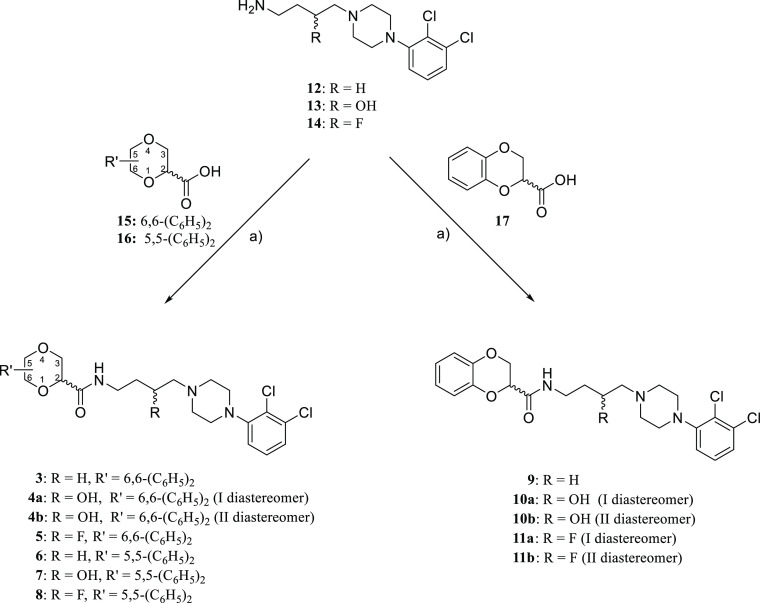
Reagents: (a) 1,1′-Carbonyldiimidazole, THF

Intermediate building blocks **12–16** were prepared according to the previously reported procedures.^32,42−44^ The novel derivatives **3–11** were prepared according to Scheme 1 by amidation of acids **15** and **16**(32) or the commercially available **17** with amines **12–14**(42−44) in the presence of 1,1′-carbonyldiimidazole in THF. The diastereomers were separated by column chromatography (**4a** and **4b**), fractional crystallization (**10a** and **10b**), or preparative thin-layer chromatography (TLC) (**11a** and **11b**) as described in the Methods section. Given the poor biological results of the separated diastereoisomers (Table 1), we did not attempt to assign their relative configuration.

**Table 1:** Affinity Values (*K*_*i*_)^a^ of **1–11** at Human D_2_-like Receptor Subtypes and of **1–3**, **6**, **9**, Clozapine, and Olanzapine at Human D_1_R, 5-HT_1A_R, 5-HT_2A_R, and 5-HT_2C_R

	*K*_*i*_ ± SEM (nM), human cloned receptors								
compd	D_1_R	D_2_R	D_3_R	D_4_R	D_2_/D_3_	D_4_/D_3_	5-HT_1A_R	5-HT_2A_R	5-HT_2C_R
1	22.9 ± 3.1^b^	12.2 ± 1.5^b^	13.1 ± 1.7^b^	8.34 ± 0.6^b^	0.9	0.6	0.66 ± 0.02^b^	52.5 ± 4.4^b^	1819 ± 165^b^
2	123 ± 11^b^	3.16 ± 1.0^b^	1.38 ± 0.07^b^	10.5 ± 0.1^b^	2	8	1.15 ± 0.03^b^	1412 ± 224^b^	9772 ± 501^b^
3	442 ± 52	342 ± 105	2.39 ± 0.69	1352 ± 449	143	566	288 ± 15	257 ± 38	642 ± 80
4a	ND^d^	39.5%^c^	164 ± 52	39.2%^c^	ND^d^	ND^d^	ND^d^	ND^d^	ND^d^
4b	ND^d^	41%^c^	77.1 ± 25.5	54.4%^c^	ND^d^	ND^d^	ND^d^	ND^d^	ND^d^
5	ND^d^	21.2%^c^	716 ± 178	19.1%^c^	ND^d^	ND^d^	ND^d^	ND^d^	ND^d^
6	700 ± 150	28.7 ± 5.4	1.58 ± 0.24	54.6 ± 7.3	18	35	9.1 ± 0.97	28 ± 6.0	90 ± 16
7	ND^d^	526 ± 28.2	23.4 ± 3.1	390 ± 46	23	17	ND^d^	ND^d^	ND^d^
8	ND^d^	1200 ± 210	295 ± 100	43.6%^c^	4	ND^d^	ND^d^	ND^d^	ND^d^
9	680 ± 160	46.4 ± 5.9	2.16 ± 0.63	74.7 ± 24.5	22	35	23.8 ± 5.2	62 ± 15	97 ± 25
10a	ND^d^	1097 ± 125	44.1 ± 8.2	282 ± 76	25	6	ND^d^	ND^d^	ND^d^
10b	ND^d^	783 ± 19	23.4 ± 2.4	244 ± 54	35	10	ND^d^	ND^d^	ND^d^
11a	ND^d^	10.6%^c^	54.8%^c^	29.4%^c^	ND^d^	ND^d^	ND^d^	ND^d^	ND^d^
11b	ND^d^	8.7%^c^	65.4%^c^	26.6%^c^	ND^d^	ND^d^	ND^d^	ND^d^	ND^d^
clozapine^e^	22.9^e^	135^e^	219^e^	46.8^e^	0.62^e^	0.21^e^	87^e^	4.07^e^	2.75^e^
olanzapine^e^	11.7^e^	21.4^e^	34.7^e^	17.8^e^	0.62^e^	0.51^e^	1514^e^	1.32^e^	3.89^e^

a *K*_i_ values were determined by competitive inhibition of [^3^H]SCH23390 binding in mouse fibroblast cells stably expressing hD_1_R; [^3^H]*N*-methylspiperone binding in HEK 293 cells stably expressing hD_2_R, hD_3_R, or hD_4_R; [^3^H]8-OH-DPAT binding in HeLa cells stably expressing h5-HT_1A_R; and [^125^I]DOI binding in HEK cells stably expressing h5-HT_2A_R or h5-HT_2C_R.

b Taken from ref (41).

c For low-affinity compounds, only the inhibition percentage of the radioligand binding at a test compound concentration of 10 μM is given. D_1_R, 5-HT_1A_R, 5-HT_2A_R, and 5-HT_2c_R data were obtained through the NIDA Addiction Treatment Discovery Program contract with Oregon Health & Science University.

d ND = not determined.

e Taken from ref (48).

The novel hybrid derivatives **3–11** were tested at hD_2_R, hD_3_R, or hD_4_R stably expressed in HEK293 cells by competition binding assays, using [^3^H]*N*-methylspiperone as the radioligand, to evaluate their D_2_-like affinity and subtype selectivity, following the previously reported procedures.^41,45,46^

The *K*_*i*_ values, calculated according to the Cheng–Prusoff equation,^47^ are shown in Table 1 along with those of the previously reported compounds **1** and **2** and the antipsychotic agents clozapine and olanzapine.

The results reveal that all the new compounds **3–11** show higher affinity for D_3_R with respect to D_2_R and D_4_R. The nature of both the linker and the SP plays a crucial role in the interaction with all the D_2_-like receptors and in the subtype–selectivity profile of the ligands. In particular, the unsubstituted butyl chain confers to the ligands the highest affinities, while the presence of a 3-hydroxy or especially the inclusion of a 3-fluoro substituent is detrimental to D_2_-like receptor binding. Indeed, compounds **3**, **6**, and **9**, each bearing an unsubstituted butyl chain, display *K*_*i*_ values significantly lower than those of the 3-hydroxybutyl compounds **4**, **7**, and **10** and, especially, of the 3-fluorobutyl derivatives **5**, **8**, and **11**. Minimal differences in affinity are observed between the 3-hydroxybutyl diastereomers **4a** and **4b** as well as **10a** and **10b**, nor between the 3-fluorobutyl diastereomers **11a** and **11b**, suggesting that the relative configuration between the stereocenters in the butyl chain and in position 2 of the 1,4-dioxane nucleus does not play a crucial role in the receptor interaction.

The nature of the SP also affects the D_3_R selectivity profiles of the ligands. Compound **3**, bearing the 6,6-diphenyl-1,4-dioxane scaffold, shows the best D_3_R selectivity profile (D_2_/D_3_ = 143 and D_4_/D_3_ = 566) compared to the 5,5-diphenyl-1,4-dioxane and the 1,4-benzodioxane derivatives **6** (D_2_/D_3_ = 18 and D_4_/D_3_ = 35) and **9** (D_2_/D_3_ = 22 and D_4_/D_3_ = 35), respectively. Compared to the already published 6,6-diphenyl-1,4-dioxane multitarget compounds **1** and **2**, the hybrid derivative **3** maintains high affinity only for D_3_R, gaining in D_3_R subtype selectivity.

The most promising hybrids (**3**, **6**, and **9**) were also evaluated for their binding affinity at D_1_R ([^3^H]SCH23390, mouse fibroblast cells), 5-HT_1A_R ([^3^H]8-OH-DPAT, HeLa cells), 5-HT_2A_R, and 5-HT_2C_R ([^125^I]DOI, HEK cells) (data were obtained through the NIDA Addiction Treatment Discovery Program contract with Oregon Health & Science University) and the results are reported in Table 1.

Interestingly, compound **3** shows selectivity for D_3_R not only over D_2_R and D_4_R but also over all other studied receptors (D_1_/D_3_ = 185, 5-HT_1A_/D_3_ = 121, 5-HT_2A_/D_3_ = 108, and 5-HT_2C_/D_3_ = 269). Compounds **6** and **9** also display negligible D_1_R affinity but significantly lower *K*_*i*_ values at the serotoninergic 5-HT_1A_R, 5-HT_2A_R, and 5-HT_2C_R subtypes and, therefore, are characterized by a more balanced 5-HT/D_2_-like multitarget profile.

*In vitro* functional assays were also conducted for derivatives **3**, **6**, and **9** at all receptors at which they showed *K*_*i*_ values < 500 nM. The data, obtained through the NIDA Addiction Treatment Discovery Program contract with Oregon Health & Science University, are reported in Table 2. The results confirm the D_3_R-preferential profile of **3**, which behaves as a partial agonist (EC_50_ = 9.8 nM), with low efficacy (36%) in the agonist mode; when tested as an antagonist, **3** shows an IC_50_ value of 38 nM (% inhibition = 82.7%). This compound is also a weak D_2_R antagonist and a 5-HT_1A_R full agonist and exhibits very low potencies at D_1_R and 5-HT_2A_R.

**Table 2:** Potency (EC_50_ or IC_50_)^a^ and Efficacy (% Stimulation or % Inhibition)^b^ Values of **3**, **6**, and **9** at D_1_R-D_4_R, 5-HT_1A_R, 5-HT_2A_R, and 5-HT_2C_R

	functional profile of 3		functional profile of 6		functional profile of 9	
receptor	EC_50_, nM (IC_50_, nM)	% stimul. (% inhib.)	EC_50_, nM (IC_50_, nM)	% stimul. (% inhib.)	EC_50_, nM (IC_50_, nM)	% stimul. (% inhib.)
**D**_**1**_ cAMP assay	(>10.000)	ND^c^	ND^c^	ND^c^	ND^c^	ND^c^
**D**_**2**_ mitogenesis assay	(139.2 ± 5.9)	(93.5)	(8.5 ± 1.3)	(95.2)	(40.0 ± 7.2)	(94.4)
**D**_**3**_ mitogenesis assay	9.8 ± 1.6	36.0	2.72	25.7	5.0 ± 1.7	34.0
**D**_**4**_ adenylate cyclase	ND^c^	ND^c^	21.7 ± 8.4	76.7	15.3 ± 1.9	73.1
**5-HT**_**1A**_ [^35^S]GTPγS binding	232 ± 19	82.7	23.9 ± 8.8	90.8	22.7 ± 0.82	96.3
**5-HT**_**2A**_ IP-1 formation	(7900 ± 9.8)	(45.4)	(650 ± 100)	(88.6)	(612 ± 58)	(86.8)
**5-HT**_**2C**_ IP-1 formation	ND^c^	ND^c^	380 ± 130	33	750 ± 280	37

a EC_50_ or IC_50_ values were from three experiments and data are presented as means ± SEM.

b The standard agonists SKF-38393 (D_1_R), quinpirole (D_2_-like subtypes), and serotonin (5-HT_1A_R, 5-HT_2A_R, and 5-HT_2C_R) were used to determine the % stimulation; the standard antagonists SCH 23390 (D_1_R), (+)-butaclamol (D_2_R), NGB 2904 (D_3_R), haloperidol (D_4_R), WAY 100,635 (5-HT_1A_R), ketanserin (5-HT_2A_R), and SB242084 (5-HT_2C_R) were used to determine the % inhibition.

c ND = not determined.

The results also highlight the interesting multitarget behavior of **6** and **9**, which are characterized by similar functional profiles at all the studied receptors: they both are efficacious D_2_R and 5-HT_2A_R antagonists with high and low potency, respectively, efficacious 5-HT_1A_R and D_4_R agonists with high potency, as well as D_3_R and 5-HT_2C_R partial agonists with high and low potency, respectively. Moreover, when tested as D_3_R antagonists, **6** and **9** show IC_50_ values of 6.6 nM (64.0% efficacy) and 40.4 nM (64.2% efficacy), respectively.

Given that combinations of D_2_R/D_3_R antagonism or partial agonism with 5-HT_1A_R agonism and 5-HT_2A_R antagonism have been demonstrated to be favorable in the management of schizophrenia^28,49^ and that D_4_R activation might ameliorate cognitive impairment associated with schizophrenia,^50^ the balanced multitarget profiles of **6** and **9** might be exploitable in the treatment of this disorder. Moreover, the partial activation of 5-HT_2C_R might contribute to the potential antipsychotic activity of these agents.^51^

**Figure 4:**
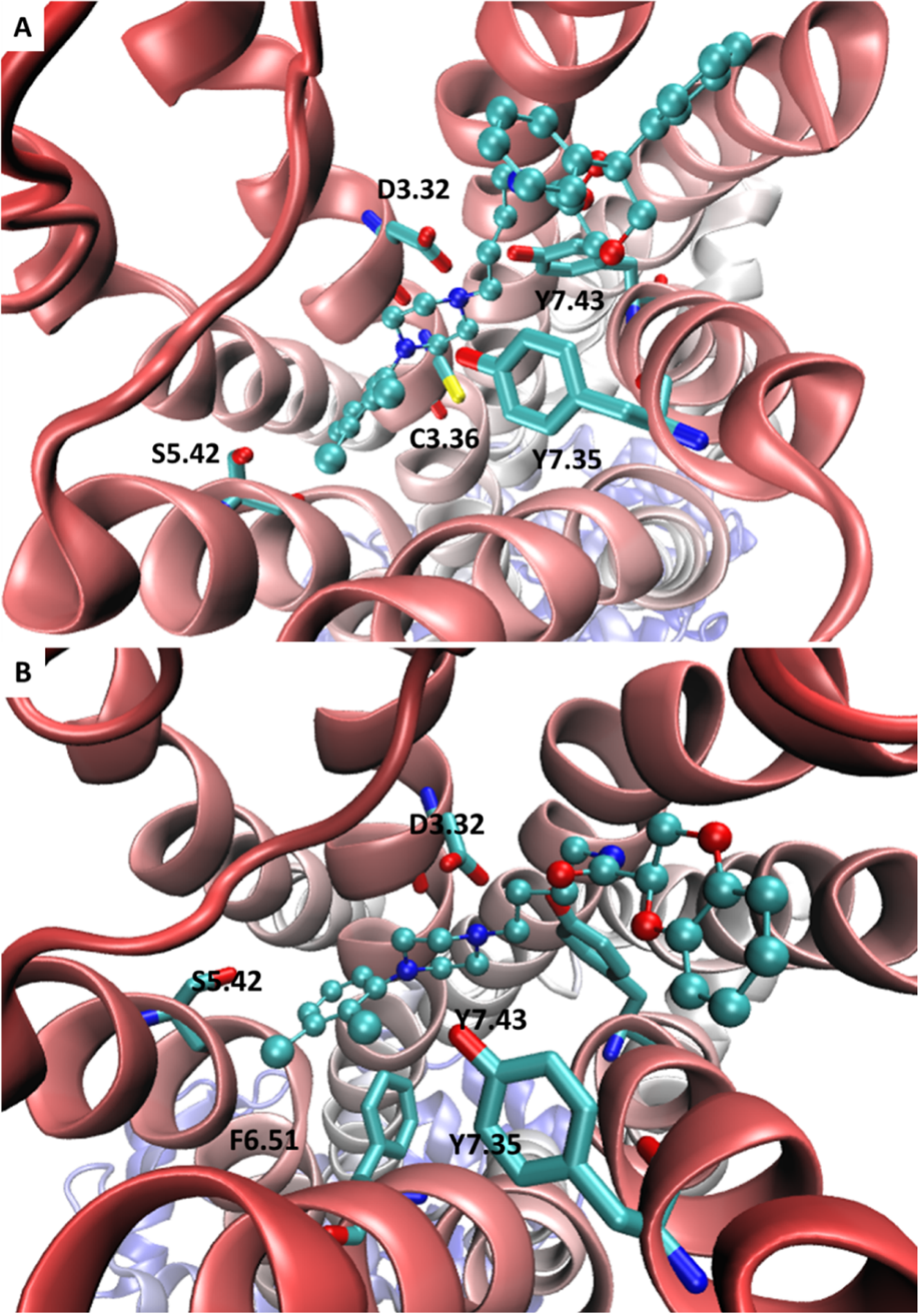
Key interactions stabilizing the putative complexes between D_3_R and **3** (A) and **9** (B) within the binding site of D_3_R.

The here reported compounds underwent docking simulations on the resolved structure of the human D_3_R in complex with eticlopride (PDB Id: 3PBL) to evaluate the main interactions stabilizing the computed complexes and to rationalize the observed differences in ligand affinity. The computed complexes of the compounds **3** and **9** also underwent 200 ns MD simulations to assess their stability and to investigate the resulting behavior of the human D_3_R structure. Figures 4 and S1 display the overall putative complexes and the main stabilizing interactions for **3** (Figures 4A and S1A) and **9** (Figures 4B and S1B) as derived by the most representative cluster from MD runs. The reported complexes reveal that the dichlorophenylpiperazine moiety of **3** and **9** assumes comparable poses stabilized by similar key interactions. In detail, the ligand ammonium head elicits an ion pair with D3.32 (Asp110) further stabilized by the H-bond with Y7.43 (Tyr373). The 2,3-dichlorophenyl ring is engaged in π–π stacking interactions with F6.51 (Phe345) and F6.52 (Phe346), while the chlorine atoms can be involved in halogen bonds with S5.42 (Ser192), S5.42 (Ser193), and H6.55 (His349). C3.36 (Cys114) can also participate in this set of contacts through a π–S interaction. While showing a greater variability, the diphenyldioxane moiety in **3**, as well as the benzodioxane ring in **9**, are harbored within the same subpocket where they mostly stabilize hydrophobic contacts. In detail, they elicit similar π–π stacking interactions with Y7.35 (Tyr365) plus hydrophobic contacts with the surrounding apolar side chains. The dioxane oxygen and the amide linker are engaged in weak H-bonds with Y7.35 (Tyr365), S7.36 (Ser366), and S7.39 (Thr369).

Figure S2A analyzes the stability of these poses as assessed by the root-mean-square deviation (rmsd) values computed for the ligand’s atoms only. The obtained rmsd profile suggests the occurrence of two possible binding modes which differ for about 2.0 Å. A visual analysis of the two corresponding complexes reveals that the differences between the two poses are focused on the arrangement of the diphenyldioxane moiety in **3** and benzodioxane ring in **9**, while the dichlorophenylpiperazine moiety of both ligands retains a significant stability during the MD simulations. Such a behavior is explainable by considering that these mobile moieties are engaged by weak interactions and thus are free to move to optimize the H-bonds stabilized by the amide linker and dioxane ring. Figure S2B shows that **3** assumes almost exclusively a binding mode which is that displayed in Figure 4 and is slightly shifted compared to that derived from docking simulations, while **9** exhibits an up and down profile with the starting pose being largely predominant. Figure S2B focuses on the rmsd profiles as computed for the backbone atoms of the human D_3_R and provides evidence of substantial stability with rmsd values almost always lower than 6.0 Å. The complex with **3** reveals a slightly greater mobility than that with **9**, which can be ascribed to its greater steric hindrance. Collectively, the overall stability of both ligands and receptor offers an encouraging confirmation for the reliability of the complexes shown in Figure 4.

Taken together, the reported computational results can explain why diphenyldioxane and benzodioxane analogues show similar affinity profiles. Indeed, these ligands stabilize an almost identical pattern of interactions with marginal differences involving the variable portion that however seems to be not so crucial for affinity, while having a key role in determining the observed selectivity profile. The D_3_R selectivity of these ligands can be explained by considering the different wideness of the more external sub-pocket that accommodates the diphenyldioxane moiety. To verify this hypothesis, docking simulations were repeated for **3** on the resolved structure of D_4_R in complex with nemonapride (PDB Id: 5WIV) by applying the same computational procedure based on the PLANTS program. In this case, the ligand is unable to assume acceptable poses in which the ammonium head contacts with D3.32 (Asp115). To further confirm the role of the pocket size in determining the selectivity of the simulated ligands, the void volume of the two binding sites was computed by using FPocket as implemented by the VEGA suite of programs^52^ and D_3_R shows a larger pocket than D_4_R (5043 *vs*. 4494 Å^3^).

Docking results indicate that fluorine and hydroxyl derivatives can stabilize comparable sets of contacts. This finding is highly expected for the fluorine substitution since a marginal structural modification cannot have such a detrimental effect on the interaction capability of the resulting ligands. Similar considerations can be drawn for the hydroxyl analogues since the putative complexes do not reveal any detrimental roles of the hydroxyl function that can take part in the contacts stabilized by the ammonium head. Hence, docking results suggest that the negative effect played by these linker substitutions should not be ascribed to their interfering role on the interaction patterns with D_3_R.

A possible explanation for the observed detrimental impact of these substitutions can be found in their effect on the basicity of the piperazine ring. An estimation of the effect of the fluorine atom on basicity can be derived by combining the available experimental p*K*_a_ value for aripiprazole, which is equal to 7.6 in 20% aqueous ethanol^53^ with the average effect of the H/F exchange in β position to an amino group which decreases its basicity of around 2.0 p*K*_a_ unit.^54^

A similar (albeit less pronounced) effect is also exerted by the hydroxyl function, which decreases the basicity of the vicinal amino groups due to the residual electronic delocalization from N to O atoms through the linking methylene groups.^55^ For β hydroxyl functions, this effect is estimated to be equal to −1.0 in the corresponding p*K*_a_ values. Hence, fluorine and hydroxyl groups have a similar effect in decreasing the basicity of the ammonium head. In both cases, the resulting p*K*_a_ value should be less than 7.0, and this means that for these derivatives, the protonated form (which is involved in receptor recognition) is no longer the most probable state at physiological pH. As detailed above, such an effect is more marked for fluorine derivatives for which the predicted p*K*_a_ value should range around 5.5, thus indicating that the protonated state is virtually absent at physiological pH. The predicted value for the hydroxyl derivatives should be around 6.5, thus suggesting that the protonated state should represent about 10–15%. Notably, the different abundances of the protonated forms for the fluorine and hydroxyl analogues are in agreement with the measured affinity values.

To further confirm this hypothesis, the computed complex for **4a** underwent a MD run with the same characteristics as described for **3** and **9**. Figure S2 also reports the rmsd profiles for the hydroxyl derivative (**4a**) and reveals a remarkable stability of the corresponding complex. As described for the previous ligands, **4a** also shows two possible binding modes even though the starting pose appears to be the most frequent one probably due to the stabilizing effect exerted by the hydroxyl group. Altogether, the performed simulations are not indicative of detrimental effects played by the hydroxyl function, and a similar consideration can also be extended to the fluorine atom and indirectly suggests that the drop in the affinity of these derivatives should be explained by considering factors that go beyond their interacting capacity with the human D_3_R.

## Conclusions

In conclusion, the biological and docking studies of the novel hybrid ligands **3–11** allowed us to get information about the role of the 1,4-benzodioxane or substituted 1,4-dioxane scaffold as the SP of bitopic compounds targeting D_3_R and to obtain novel D_3_R-selective or multitarget agents. An unsubstituted butyl chain between the pharmacophores characterizes the highest affinity compounds at D_3_R: **3**, **6**, and **9**. In particular, the 6,6-diphenyl-1,4-dioxane derivative **3** showed a D_3_R preferential profile, behaving as a low-efficacy (36%) partial agonist/antagonist. An interesting multitarget profile has been highlighted for compounds **6** and **9**, both behaving as efficacious D_2_R and 5-HT_2A_R antagonists with high and low potency, respectively, efficacious 5-HT_1A_R and D_4_R agonists with high potency, as well as D_3_R and 5-HT_2C_R partial agonists with high and low potency, respectively. Such a profile might be favorable for novel antipsychotic agents. However, further studies will be needed to support the therapeutic potential of these compounds for the treatment of schizophrenia.

## Methods

### Chemistry

#### General Procedures

Flash column chromatography was performed using silica gel (EMD Chemicals, Inc.; 230–400 mesh, 60 Å). Eluting solvents are described for each compound. ^1^H NMR (400 MHz) and ^13^C NMR (100 MHz) spectra were acquired using a Varian Mercury Plus 400 spectrometer. Chemical shifts are reported in parts per million (ppm) and referenced according to a deuterated solvent for ^1^H spectra (CDCl_3_, 7.26) and ^13^C spectra (CDCl_3_, 77.2). Combustion analysis was performed by Atlantic Microlab, Inc. (Norcross, GA), and the reported values agree within 0.4% of calculated values. Melting points were determined using a Thomas–Hoover melting point apparatus and are uncorrected. Anhydrous solvents were purchased from Aldrich and used without further purification, except for THF, which was freshly distilled from sodium-benzophenone ketyl. All other chemicals and reagents were purchased from Aldrich Chemical Co. On the basis of NMR, GC–MS, and combustion analysis data, all final compounds are >95% pure.

##### *N*-(4-(4-(2,3-Dichlorophenyl)piperazin-1-yl)butyl)-6,6-diphenyl-1,4-dioxane-2-carboxamide (**3**)

1,1′-Carbonyldiimidazole (0.18 g; 1.10 mmol) was added to a solution of **15**(32) (0.30 g; 1.10 mmol) in THF (10 mL). The reaction was stirred at room temperature for 2 h. Amine **12**(44) (0.33 g; 1.10 mmol) was added dropwise to the cooled solution (0 °C). The reaction was allowed to warm to room temperature and then stirred for 3 h. The solvent was removed under vacuum, and the residue was diluted in CHCl_3_ and washed with NaHCO_3_-saturated aqueous solution. The organic phase was dried over anhydrous Na_2_SO_4_, filtered, and concentrated under vacuum. The crude compound was purified by column chromatography eluting with EtOAc/CHCl_3_ (5:5) to afford compound **3** as an oil in 80% yield. ^1^H NMR (400 MHz, CDCl_3_): δ 7.66–6.85 (m, 13H, ArH), 4.63 (d, *J* = 32.49 Hz, 1H, dioxane), 4.18 (dd, *J* = 9.74, 9.75 Hz, 1H, dioxane), 3.79 (d, *J* = 12.07 Hz, 1H, dioxane), 3.62–3.43 (m, 2H, dioxane), 3.04 (m, 5H, CH_2_N, CH_2_NCO and NH), 2.65–2.39 (m, 8H, piperazine), 1.63 (m, 2H, CH_2_), 1.51 (m, 2H, CH_2_). ^13^C NMR (100 MHz, CDCl_3_): δ 25.40, 27.73, 39.25, 40.90, 51.33, 51.83, 74.91, 84.23, 85.10, 90.21, 117.64, 123.98, 126.26, 127.18, 128.18, 129.25, 133.34, 139.62, 150.11, 172.71. The free base was transformed into the corresponding oxalate salt, which was crystallized from 2-PrOH (m.p. 107–108 °C). Anal. calcd for C_31_H_35_Cl_2_N_3_O_3_·C_2_H_2_O_4_: C, 60.19%, H, 5.66%, N, 6.38%, found; C, 60.34%, H, 5.51%, N, 6.52%.

##### *N*-(4-(4-(2,3-Dichlorophenyl)piperazin-1-yl)-3-hydroxybutyl)-6,6-diphenyl-1,4-dioxane-2-carboxamide (**4a** and **4b**)

These compounds were prepared following the procedure described for compound **3**, starting from **15** and **13**. The crude mixture of diastereomers was purified by column chromatography eluting with EtOAc/MeOH (99:1). The diastereomer **4a** eluted first as an oil in 32% yield. ^1^H NMR (400 MHz, CDCl_3_): δ 7.71–6.95 (m, 13H, ArH), 4.61 (d, *J* = 12.50 Hz, 1H, dioxane), 4.20 (m, 1H, dioxane), 3.95–3.25 (m, 4H, CHOH, dioxane), 3.10 (m, 7H, piperazine, NCH_2_, CH_2_N, NH and OH), 2.85 (m, 2H, piperazine), 2.62 (m, 3H, piperazine), 2.48 (m, 2H, piperazine), 1.79–1.60 (m, 2H, CH_2_). The free base was transformed into the corresponding oxalate salt, which was crystallized from 2-PrOH (m.p. 146–147 °C). ^13^C NMR (100 MHz, DMSO): δ 36.43, 45.48, 51.15, 56.06, 61.68, 69.13, 71.91, 83.23, 86.62, 93.12, 117.64, 121.98, 126.22, 127.18, 128.18, 129.22, 133.34, 140.62, 150.01, 162.71. Anal. calcd for C_31_H_35_Cl_2_N_3_O_4_·C_2_H_2_O_4_: C, 58.76%, H, 5.53%, N, 6.23%, found C, 58.49%, H, 5.59%, N, 6.11%. The second fraction was the diastereomer **4b** as an oil in 20% yield. ^1^H NMR (400 MHz, CDCl_3_): δ 7.49–6.95 (m, 13H, ArH), 3.79 (d, *J* = 12.50 Hz, 1H, dioxane), 4.66 (d, *J* = 12.12 Hz, 1H, dioxane), 4.24 (m, 1H, dioxane), 4.09 (dd, *J* = 2.74, 3.51 Hz, 1H, dioxane), 3.48 (m, 2H, CHOH and dioxane), 3.08 (m, 8H, piperazine, CH_2_N, CH_2_N, NH and OH), 2.81 (m, 2H, piperazine), 2.58 (m, 2H, piperazine), 2.39 (m, 2H, piperazine), 1.63–1.51 (m, 2H, CH_2_). The free base was transformed into the corresponding oxalate salt, which was crystallized from 2-PrOH (m.p. 122–123 °C). ^13^C NMR (100 MHz, DMSO): δ 36.38, 45.58, 51.12, 56.08, 61.48, 69.13, 75.91, 83.23, 86.62, 93.12, 117.64, 123.98, 126.22, 127.18, 128.18, 129.25, 133.34, 139.62, 150.11, 172.71. Anal. calcd for C_31_H_35_Cl_2_N_3_O_4_·C_2_H_2_O_4_: C, 58.76%, H, 5.53%, N, 6.23%, found; C, 58.55%, H, 5.63%, N, 6.04%.

##### *N*-(4-(4-(2,3-Dichlorophenyl)piperazin-1-yl)-3-fluorobutyl)-6,6-diphenyl-1,4-dioxane-2-carboxamide (**5**)

This compound was prepared following the procedure described for compound **3**, starting from **15** and **14**. The crude compound was purified by column chromatography eluting with CHCl_3_/acetone (8:2) to afford diastereomer **5** as an oil in 41% yield. ^1^H NMR (400 MHz, CDCl_3_): δ 7.49–6.95 (m, 13H, ArH), 4.80 (m, 1H, CHF), 4.66 (d, *J* = 12.50 Hz, 1H, dioxane), 4.23 (dd, *J* = 3.52, 3.52 Hz, 1H, dioxane), 4.10 (dd, *J* = 3.12, 3.52 Hz, 1H, dioxane), 3.79 (d, *J* = 12.50 Hz, 1H, dioxane), 3.41 (m, 3H, CH_2_N and dioxane), 3.10 (m, 5H, piperazine and NCH_2_), 2.75 (m, 6H, piperazine and NH), 1.60–1.29 (m, 2H, CH_2_). ^19^F NMR (376 MHz, CDCl_3_/CFCl_3_): δ −183.42 to −183.98 (m, 1F). The free base was transformed into the corresponding oxalate salt, which was crystallized from EtOH (m.p. 100–101 °C). ^13^C NMR (100 MHz, DMSO): δ 33.51, 34.80, 51.31, 51.88, 61.10, 74.90, 84.23, 85.00, 89.81, 90.20, 117.64, 121.98, 126.22, 127.18, 128.18, 129.22, 133.34, 140.62, 150.01, 162.71. Anal. calcd for C_31_H_34_Cl_2_FN_3_O_3_·C_2_H_2_O_4_: C, 58.58%, H, 5.36%, N, 6.21%, found; C, 58.28%, H, 5.44%, N, 6.10%.

##### *N*-(4-(4-(2,3-Dichlorophenyl)piperazin-1-yl)butyl)-5,5-diphenyl-1,4-dioxane-2-carboxamide (**6**)

This compound was prepared following the procedure described for compound **3**, starting from **16**(32) and **12** to afford **6** as an oil in 65% yield. ^1^H NMR (400 MHz, CDCl_3_): δ 7.66–6.85 (m, 13H, ArH)1.56 (m, 2H, CH_2_), 4.87 (d, *J* = 29.31 Hz, 1H, dioxane), 3.64–3.24 (m, 4H, dioxane), 3.11 (m, 5H, NCH_2_, CH_2_N and NH), 2.69–2.35 (m, 8H, piperazine), 1.72 (m, 2H, CH_2_). ^13^C NMR (100 MHz, CDCl_3_): δ 25.40, 27.73, 39.25, 40.90, 51.33, 51.85, 69.13, 82.21, 90.04, 92.96, 117.64, 123.98, 126.22, 127.18, 128.26, 129.29, 133.34, 139.68, 150.11, 172.71. The free base was transformed into the corresponding oxalate salt, which was crystallized from EtOH (m.p. 94–95 °C). Anal. calcd for C_31_H_35_Cl_2_N_3_O_3_·C_2_H_2_O_4_: C, 60.19%, H, 5.66%, N, 6.38%, found; C, 59.99%, H, 5.48%, N, 6.60%.

##### *N*-(4-(4-(2,3-Dichlorophenyl)piperazin-1-yl)-3-hydroxybutyl)-5,5-diphenyl-1,4-dioxane-2 Carboxamide (**7**)

This compound was prepared following the procedure described for compound **3**, starting from **16** and **13**.^43^ The crude compound was purified by column chromatography eluting with EtOAc/CHCl_3_/MeOH (5:5:1) to afford diastereomer **7** as an oil in 32% yield. ^1^H NMR (400 MHz, CDCl_3_): δ 7.66–6.85 (m, 13H, ArH), 4.89 (m, 1H, dioxane), 3.93–3.59 (m, 4H, dioxane), 3.31 (m, 1H, CHOH), 3.08 (m, 6H, NCH_2_, CH_2_N, NH and OH), 2.92–2.31 (m, 8H, piperazine), 1.65–1.46 (m, 2H, CH_2_). ^13^C NMR (100 MHz, CDCl_3_): δ 36.33, 45.68, 51.82, 56.08, 61.48, 69.13, 75.91, 83.23, 86.62, 93.12, 117.64, 123.98, 126.22, 127.18, 128.26, 129.22, 133.34, 139.68, 150.11, 172.71. The free base was transformed into the corresponding oxalate salt, which was crystallized from MeOH (m.p. 132–133 °C). Anal. calcd for C_31_H_35_Cl_2_N_3_O_4_·C_2_H_2_O_4_: C, 58.76%, H, 5.53%, N, 6.23%, found, C, 59.01%, H, 5.40%, N, 6.35%.

##### *N*-(4-(4-(2,3-Dichlorophenyl)piperazin-1-yl)-3-fluorobutyl)-5,5-diphenyl-1,4-dioxane-2-carboxamide (**8**)

This compound was prepared following the procedure described for compound **3**, starting from **16** and **14**.^42^ The crude compound was purified by column chromatography eluting with CHCl_3_/acetone (8:2) to afford diastereomer **8** as an oil in 64% yield. ^1^H NMR (400 MHz, CDCl_3_): δ 7.49–6.95 (m, 13H, ArH), 4.85 (m, 2H, CHF and dioxane), 4.30 (m, dioxane), 3.78 (m, 1H, dioxane), 3.58 (m, 2H, CH_2_N), 3.01 (m, 4H, piperazine and NCH_2_), 2.68 (m, 3H, piperazine and NH), 2.48 (m, 2H, piperazine), 2.29 (m, 2H, piperazine), 1.63–1.45 (m, 2H, CH_2_). The free base was transformed into the corresponding oxalate salt, which was crystallized from 2-PrOH (m.p. 126–127 °C). ^13^C NMR (100 MHz, DMSO): δ ppm 33.43, 34.78, 51.29, 51.88, 61.10, 69.13, 82.23, 89.84, 90.08, 93.10, 117.64, 121.98, 126.22, 127.18, 128.18, 129.33, 133.34, 140.62, 150.01, 162.71. ^19^F NMR (376 MHz, DMSO): δ ppm −181.12 to −181.58 (m, 1F). Anal. calcd for C_31_H_34_Cl_2_FN_3_O_3_·C_2_H_2_O_4_: C, 58.58%, H, 5.36%, N, 6.21%, found; C, 58.33%, H, 5.53%, N, 6.06%.

##### *N*-(4-(4-(2,3-Dichlorophenyl)piperazin-1-yl)butyl)-2,3-dihydrobenzo[*b*][1,4]dioxine-2-carboxamide (**9**)

This compound was prepared following the procedure described for compound **9**, starting from **17** and **12**. The crude compound was purified by column chromatography eluting with EtOAc/CHCl_3_ (5:5) to afford compound **9** as an oil in 58% yield. ^1^H NMR (400 MHz, CDCl_3_): δ 7.18–6.90 (m, 7H, ArH), 4.68 (dd, *J* = 7.43, 7.03 Hz, 1H, dioxane), 4.54 (dd, *J* = 2.73, 2.73 Hz, 1H, dioxane), 4.19 (dd, *J* = 7.03, 7.43 Hz, 1H, dioxane), 3.39 (m, 2H, NCH_2_), 3.11 (m, 3H, CH_2_N and NH), 2.63 (m, 4H, piperazine), 2.42 (m, 4H, piperazine), 1.60 (m, 4H, CH_2_CH_2_). The free base was transformed into the corresponding oxalate salt, which was crystallized from 2-PrOH (m.p. 167–168 °C). ^13^C NMR (100 MHz, DMSO): δ 21.29, 26.63, 38.24, 48.72, 51.73, 55.86, 65.29, 73.03, 117.44, 117.79, 120.22, 121.92, 122.01, 125.55, 126.51, 129.03, 133.14, 142.61, 143.48, 150.28, 164.73, 167.22. Anal. calcd for C_23_H_27_Cl_2_N_3_O_3_·C_2_H_2_O_4_: C, 54.16%, H, 5.27%, N, 7.58%, found; C, 54.01%, H, 5.34%, N, 7.32%.

##### *N*-(4-(4-(2,3-Dichlorophenyl)piperazin-1-yl)-3-hydroxybutyl)-2,3-dihydrobenzo[*b*][1,4]dioxine-2-carboxamide (**10a** and **10b**)

These compounds were prepared following the procedure described for compound **3**, starting from **17** and **13**. The crude compound was purified by column chromatography eluting with CHCl_3_/CH_3_OH (95:5) to afford the diastereomeric mixture **10a**/**10b** as an oil in 45% yield. ^1^H NMR (400 MHz, CDCl_3_): δ ppm 1.51–1.70 (m, 2H, CH_2_), 2.39 (m, 2H, piperazine), 2.56 (m, 2H, piperazine), 2.78 (m, 2H, piperazine), 3.04 (m, 4H, piperazine, NH and OH), 3.37 (m, 2H, CH_2_N), 3.63 (m, 2H, NCH_2_), 3.80 (m, 1H, CHOH), 4.22 (m, 1H, dioxane), 4.49 (m, 1H, dioxane), 4.67 (dd, *J* = 6.64, 7.03, 1H, dioxane), 6.90–7.12 (m, 7H, ArH). The free bases were transformed into the corresponding oxalate salts, and the diastereomers were separated by crystallization from 2-PrOH. Diastereomer **10a** (m.p. 88–89 °C): ^1^H NMR (400 MHz, DMSO): δ 8.20 (m, 1H, ArH), 7.31 (m, 2H, ArH), 7.16 (m, 1H, ArH), 6.95 (m, 1H, ArH), 6.81 (m, 2H, ArH), 4.77 (dd, *J* = 5.85, 5.86 Hz, 1H, dioxane), 4.31 (dd, *J* = 3.10, 2.25 Hz, 1H, dioxane), 4.20 (dd, *J* = 6.30, 5.86 Hz, 1H, dioxane), 3.82 (m, 1H, CHOH), 2.43 (m, 2H, piperazine), 3.19 (m, 10H, piperazine, NCH_2_, CH_2_N, NH and OH), 3.01 (m, 1H, piperazine), 2.90 (m, 1H, piperazine), 1.45–1.55 (m, 2H, CH_2_). ^13^C NMR (100 MHz, DMSO): δ 25.92, 35.13, 35.72, 48.64, 52.46, 54.19, 61.79, 67.76, 73.04, 90.79, 105.00, 117.74, 120.22, 121.99, 122.06, 125.14, 126.52, 129.05, 133.49, 142.60, 143.48, 150.37, 163.82, 167.34. Anal. calcd for C_23_H_27_Cl_2_N_3_O_4_·C_2_H_2_O_4_: C, 52.64%, H, 5.12%, N, 7.37%, found; C, 52.37%, H, 5.30%, N, 7.51%. Diastereomer **10b** (m.p. 99–100 °C). ^1^H NMR (400 MHz, DMSO): δ 8.20 (m, 1H, ArH), 7.37 (m, 2H, ArH), 7.20 (m, 1H, ArH), 6.95 (m, 1H, ArH), 6.81 (m, 2H, ArH), 4.80 (dd, *J* = 5.85, 5.86 Hz, 1H, dioxane), 4.31 (d, *J* = 11.26 Hz, 1H, dioxane), 4.22 (dd, *J* = 5.41, 5.86 Hz, 1H, dioxane), 3.84 (m, 1H, CHOH), 3.10 (m, 11H, piperazine, NCH_2_, CH_2_N, NH and OH), 2.95 (m, 1H, piperazine), 2.43 (m, 2H, piperazine), 1.53 (m, 2H, CH_2_). ^13^C NMR (100 MHz, DMSO): δ 15.57, 35.08, 35.62, 48.28, 52.56, 61.35, 63.10, 65.32, 73.01, 102.34, 106.67, 117.45, 117.75, 120.22, 122.03, 125.63, 126.48, 129.05, 133.14, 142.60, 143.48, 150.17, 163.10, 167.35. Anal. calcd for C_23_H_27_Cl_2_N_3_O_4_·C_2_H_2_O_4_: C, 52.64%, H, 5.12%, N, 7.37%, found; C, 52.45%, H, 5.28%, N, 7.56%.

##### *N*-(4-(4-(2,3-Dichlorophenyl)piperazin-1-yl)-3-fluorobutyl)-2,3-dihydrobenzo[*b*][1,4]dioxine-2-carboxamide (**11a** and **11b**)

These compounds were prepared following the procedure described for compound **3**, starting from **17** and **14** to afford the diastereomeric mixture **11a**/**11b** as an oil in 36% yield. ^1^H NMR (400 MHz, CDCl_3_): δ 7.18 (m, 2H, ArH), 6.78–6.99 (m, 5H, ArH), 4.92 (m, 1H, CHF), 4.70 (m, 1H, 2-CH dioxane), 4.49 (m, 1H, 3-CH dioxane), 4.21 (m, 1H, 3-CH dioxane, diastereomeric ratio 50:50), 3.49 (m, 2H, CH_2_N), 3.04 (m, 4H, piperazine and NCH_2_), 2.63–2.88 (m, 6H, piperazine and NH), 2.18 (m, 1H, piperazine), 1.90 (m, 2H, CH_2_). The diastereomers **11a** and **11b** were separated by preparative TLC eluting with CHCl_3_/CH_3_OH (95:5). ^19^F NMR (376 MHz, CDCl_3_/CFCl_3_): δ ppm −182.18 to −182.43 (m, 1F, diastereomer **11a**), −182.60 to −182.98 (m, 1F, diastereomer **11b**). The free bases were transformed into the corresponding oxalate salts, which were crystallized from 2-PrOH. Diastereomer **11a** (m.p. 200–201 °C). Anal. calcd for C_23_H_26_Cl_2_FN_3_O_3_·C_2_H_2_O_4_: C, 52.46%, H, 4.93%, N, 7.34%, found; C, 52.67%, H, 5.08%, N, 7.25%. C, H, N. Diastereomer **11b** (m.p. 180–182 °C). Anal. calcd for C_23_H_26_Cl_2_FN_3_O_3_·C_2_H_2_O_4_: C, 52.46%, H, 4.93%, N, 7.34%, found; C, 52.22%, H, 5.03%, N, 7.22%.

### Receptor Binding Studies

#### Radioligand Binding Assays at Human D_2_R, D_3_R, and D_4_R

Binding at dopamine D_2_-like receptors was determined using previously described methods.^41,45,46^ Membranes were prepared from HEK293 cells stably expressing human D_2_, D_3_, or D_4_, grown in a 50:50 mix of Dulbecco’s minimal essential medium (DMEM) and Ham’s F12 culture media, supplemented with 20 mM HEPES, 2 mM l-glutamine, 0.1 mM nonessential amino acids, 1× antibiotic/antimycotic, 10% heat-inactivated fetal bovine serum, and 200 μg/mL hygromycin (Life Technologies, Grand Island, NY) and kept in an incubator at 37 °C and 5% CO_2_. Upon reaching 80–90% confluency, the cells were harvested using premixed Earle’s Balanced Salt Solution (EBSS) with 5 μM EDTA (Life Technologies) and centrifuged at 3000 rpm for 10 min at 21 °C. The supernatant was removed, and the pellet was resuspended in 10 mL hypotonic lysis buffer (5 mM MgCl_2_, 5 mM Tris, pH 7.4 at 4 °C) and centrifuged at 20,000 rpm for 30 min at 4 °C. The pellet was then resuspended in fresh EBSS buffer made from 8.7 g/L Earle’s Balanced Salts without phenol red (US Biological, Salem, MA), 2.2 g/L sodium bicarbonate, pH to 7.4. A Bradford protein assay (Bio-Rad, Hercules, CA) was used to determine the protein concentration, and membranes were diluted to 500 μg/mL and stored at −80 °C for later use. Radioligand competition binding experiments were conducted using thawed membranes. Test compounds were freshly dissolved in 30% DMSO and 70% H_2_O to a stock concentration of 100 μM. To assist the solubilization of free-base compounds, 10 μL of glacial acetic acid was added along with DMSO. Each test compound was then diluted into 13 half-log serial dilutions using 30% DMSO vehicle; the final test concentrations ranged from 10 μM to 10 pM. The previously frozen membranes were diluted in fresh EBSS to a 100 μg/mL (for D_2_ or D_3_) or 200 μg/mL (D_4_) stock for binding. Radioligand competition experiments were conducted in glass tubes containing 300 μL of fresh EBSS buffer, 50 μL of diluted test compound, 100 μL of membranes (10 μg of total protein for D_2_ or D_3_ and 20 μg of total protein for D_4_), and 50 μL of [^3^H]*N*-methylspiperone (0.4 nM final concentration; PerkinElmer). Nonspecific binding was determined using 10 μM butaclamol (Sigma-Aldrich, St. Louis, MO), and total binding was determined with the 30% DMSO vehicle. All compound dilutions were tested in triplicate and the reaction was incubated for 1 h at room temperature. The reaction was terminated by filtration through Whatman GF/B filters, presoaked for 1 h in 0.5% polyethylenimine, using a Brandel R48 filtering manifold (Brandel Instruments, Gaithersburg, MD). The filters were washed three times with 3 mL of ice-cold EBSS buffer and transferred to scintillation vials. CytoScint liquid scintillation cocktail (3 mL, MP Biomedicals, Solon, OH) was added, and vials were counted using a PerkinElmer Tri-Carb 2910 TR liquid scintillation counter (Waltham, MA).

#### Radioligand Binding Assay at Human D_1_R

Mouse fibroblast cells expressing the human D_1_R at high density (LhD_1_ cells) are used. The cells are grown to confluence in DMEM containing 10% FetalClone1 serum (FCS, HyClone), 0.05% penicillin–streptomycin (pen-strep), and 400 μg/mL of Geneticin (G418). Three confluent 150 mm plates yield enough membranes for three assay plates with ∼10–15 μg protein per well. The cells from three 150 mm plates are scraped and centrifuged at 500×*g* for 5 min. The pellet is overlaid with 2 mL assay buffer (50 mM Tris–HCl containing 120 mM NaCl, 5 mM KCl, 2 mM CaCl_2_, and 1 mM MgCl_2_) and frozen at −70 °C. On the day of experiment, the pellet is homogenized in 30 mL assay buffer with a polytron. Cell homogenate (100 μL) is added to wells containing 800 μL of test drug or buffer. After a 10 min preincubation, 100 μL of [^3^H]SCH-23390 (0.18 nM final concentration) is added. The plates are incubated at 25 C for 60 min. The reaction is terminated by filtration using a Tomtec 96 well harvester, and the radioactivity on the filters is determined using a PerkinElmer microbeta scintillation counter. Nonspecific binding is determined with 1 μM SCH-23390.

#### Radioligand Binding Assay at Human 5-HT_1A_R

A human cell line (HeLa) stably transfected with genomic clone G-21 coding for the human 5-HT_1A_R was used. Cells were grown as monolayers in Dulbecco’s modified Eagle’s medium supplemented with 10% fetal calf serum and gentamycin (100 μg/mL) under 5% CO_2_ at 37 °C. Cells were detached from the growth flask at 95% confluency by a cell scraper and were lysed in ice-cold Tris (5 mM) and EDTA buffer (5 mM, pH 7.4). Homogenates were centrifuged for 20 min at 40000*g*, and pellets were resuspended in a small volume of ice-cold Tris/EDTA buffer (above) and immediately frozen and stored at -70 °C until use. On the day of experiment, cell membranes (80–90 μg of protein) were resuspended in binding buffer (50 mM Tris, 2.5 mM MgCl_2_, and 10 mM pargyline, pH 7.4). Membranes were incubated in a final volume of 0.32 mL for 30 min at 30 °C with 1 nM [^3^H]8-OH-DPAT in the absence or presence of various concentrations of competing drugs (1 pM to 1 μM); each experimental condition was performed in triplicate. Nonspecific binding was determined in the presence of 10 μM 5-HT.

#### Radioligand Binding Assay at Human 5-HT_2A_R and 5-HT_2C_R

Human embryonic kidney cells expressing human 5HT_2A_R (HEK-h5HT_2A_) or human 5HT_2C_R (HEK-h5HT_2C_) are used. The cells are grown until confluent on 15 cm plates. The medium is removed, and the cells are washed with phosphate-buffered saline (PBS), scraped into 2 mL PBS, and frozen at −20 °C until needed. Cell suspension is thawed, 10 mL assay buffer (50 mM Tris, pH 7.4 at 37 °C, with 0.1% ascorbic acid and 5 mM CaCl_2_) is added per plate of cells, and polytronned at setting 6 for 5 s. The homogenate is centrifuged at 15,500 rpm for 20 min. To minimize the residual 5HT concentration, the pellet is resuspended in buffer, polytronned, and centrifuged as mentioned above. The final pellet is resuspended in 2 mL buffer/plate of cells. The binding assay includes 50 μL drug, 5-HT or buffer, 50 μL cell homogenate, 50 μL [^125^I]DOI (∼0.1 nM), and buffer in a final volume of 250 μL. Specific binding is defined as the difference between total binding and binding in the presence of 10 μM 5HT. The reaction is incubated for 1 h at 37 °C and terminated by filtration through Wallac A filter mats presoaked in 0.05% polyethylenimine using a Tomtec 96-well harvester. The radioactivity remaining on filters is determined using a Wallac betaplate reader.

### Functional Assays

#### Adenylate Cyclase/cAMP Functional Assay

Mouse glioma cells expressing the monkey D_1_R at low density (C6D1 low-D_1_-density cells) are used. The cells are grown on 48-well plates in DMEM containing 10% FCS, 0.05% pen-strep, and 2 g/mL of puromycin. When the wells reach 80–90% confluency, DMEM is removed, and each well is rinsed once with 0.5 mL of EBSS buffer (116.4 mM NaCl, 5.4 mM KCl, 1.2 mM Na_2_HPO_4_, 1.3 mM CaCl_2_, 1.2 mM MgSO_4_, 15 mM HEPES, and 10 mM glucose, pH 7.4 at room temperature) containing 0.1% ascorbic acid, 2% bovine calf serum (BCS), and 0.11% 3-isobutyl-1-methyl-xanthine (IBMX), an inhibitor of cAMP phosphodiesterase. The cell number of C6D1 low-D_1_-density cells per 48-well plate is determined. Two hundred thousand cells per well is optimal for the use of the cyclic AMP EIA kit (Cayman Chemical). The 48-well plates are preincubated for 20 min at 37 °C with EBSS buffer alone and then incubated for an additional 20 min with the test compound diluted in EBSS buffer (final volume 1 mL) in triplicate. The EBSS buffer is removed and 0.05 mL of 3% TCA is added to each well. After 1 h, the supernatant is diluted 1:50, and 50 μL of diluted supernatant is added to the cyclic AMP EIA test plate in a final assay volume of 200 μL/well. The assay is incubated for 18 h at 4 °C. The supernatant is aspirated, and each well is rinsed five times with 300 μL of EIA buffer. The developing reagent (200 μL, Ellman’s) is added to each well and incubated at room temperature for 2 h while gently rotating. The plate is read in a microplate spectrophotometer at 405 nM (BioRad Benchmark Plus). The linearity of signal is observed at cAMP amounts up to ∼100 pg.

#### Mitogenesis Functional Assay

CHOp-D2 and CHOp-D3 cells are maintained in α-MEM with 10% fetal bovine serum (FBS, Atlas Biologicals), 0.05% pen-strep, and 400 μg/mL of G418. To measure D_2_R or D_3_R stimulation of mitogenesis (agonist assay) or inhibition of quinpirole stimulation of mitogenesis (antagonist assay), CHOp-D2 or CHOp-D3 cells, respectively, are seeded in a 96-well plate at a concentration of 5000 cells/well. The cells are incubated at 37 °C in α-MEM with 10% FBS. After 48–72 h, the cells are rinsed twice with serum-free α-MEM and incubated for 24 h at 37 °C. Serial dilutions of test compounds are made by the Biomek robotics system in serum-free α-MEM. In the functional assay for agonists, the medium is removed and replaced with 100 μL of test compound in serum-free α-MEM. In the antagonist assay, the serial dilution of the putative antagonist test compound is added in 90 μL (1.1× of final concentration) and 300 nM quinpirole (30 nM final) is added in 10 μL. After another 24 h (D_2_) or 16 h (D_3_) incubation at 37 °C, 0.25 μCi of [^3^H]thymidine in αMEM supplemented with 10% FBS is added to each well, and the plates are further incubated for 2 h at 37 °C. The cells are trypsinized by addition of 10× trypsin solution (1% trypsin in calcium–magnesium-free phosphate-buffered saline), and the plates are filtered and counted as usual. Quinpirole is run each day as an internal control, and dopamine is included for comparative purposes.

#### Adenylate Cyclase/cAMP Functional Assay

HEK-D4.4-AC1 cells are grown to confluency on 150 mM plates. The cells are plated at a density of 375,000 cells per well in 48-well plates in DMEM supplemented with 5% FetalClone, 5% BCS, and pen-strep. After ∼36 h, the medium is changed to DMEM supplemented with 10% charcoal-stripped FetalClone and pen-strep. The medium is removed ∼18 h later. For agonist assays, 0.8 mL of EBSS (116 mM NaCl, 22 mM glucose, 15 mM HEPES, 8.7 mM NaH_2_PO_4_, 5.4 mM KCl, 1.3 mM CaCl_2_, 1.2 mM MgSO_4_, 1 mM ascorbic acid, 0.5 mM IBMX [3-isobutyl-1-methyl-xanthine], and 2% BCS, pH 7.4 at 37 °C) is added, cells are incubated for 20 min, agonists are added, and, after a 20 min incubation, 10 μM of forskolin is added in a final volume of 1 mL. For antagonists, 0.7 mL of EBSS is added, cells are incubated for 10 min, antagonists are added, cells are incubated for 10 min, 2 nM quinpirole is added, and after a 20 min incubation, 10 μM forskolin is added in a final volume of 1 mL. For all conditions, after a 20 min incubation with forskolin, the reaction is terminated by aspiration of the buffer, and 0.1 mL of trichloroacetic acid is added. The plates are incubated for 2 h on a rotator. Adenylate cyclase activity is measured using a cyclic AMP EIA kit (Cayman). Aliquots (9 μL) of each well are diluted to 200 μL with EIA buffer from the kit, and 50 μL of dilution is added to the EIA plate. After addition of tracer and monoclonal antibody, the EIA plates are incubated for 18 h at 4 °C. The reaction is aspirated, the plates are washed with 5 × 300 μL wash buffer, and Ellman’s reagent is added. After a 2 hour incubation in the dark on a rotator, the plates are read at 410 nm. Basal cAMP is subtracted from all values. D_4_R agonists inhibit forskolin-stimulated cAMP formation, and maximal inhibition is defined with 1 μM quinpirole. Data normalized to percent forskolin stimulation are shown in the graph. The maximal effect is normalized to maximal effect of quinpirole in the tables. For antagonists, maximal reversal of inhibition of cAMP formation is defined with 10 μM haloperidol.

#### [^35^S]GTPγS Binding Assay

The effects of the various compounds tested on [^35^S]GTPγS binding in HeLa cells expressing the recombinant human 5-HT_1A_R were evaluated according to the method of Stanton and Beer [41] with minor modifications. The stimulation experiments are as follows: Cell membranes (50–70 μg of protein) were resuspended in buffer containing 20 mM HEPES, 3 mM MgSO_4_, and 120 mM NaCl (pH 7.4). The membranes were incubated with 30 μM GDP and various concentrations (from 0.1 nM to 10 μM) of test drugs or 8-OH-DPAT (reference curve) for 20 min at 30 °C in a final volume of 0.5 mL. The samples were transferred to ice, [^35^S]GTPγS (200 pM) was added, and the samples were incubated for further 30 min at 30 °C. The preincubation with both agonist and antagonist, before initiating the [^35^S]GTPγS binding, ensures that agonist and antagonist are at equilibrium. Nonspecific binding was determined in the presence of 10 mM GTPγS. Incubation was stopped by the addition of ice-cold HEPES buffer and rapid filtration on Unifilter B filters (PerkinElmer) using a Filtermate cell harvester (Packard). The filters were washed with ice-cold Hepes buffer, and the radioactivity retained on the filters was determined by a TopCount, PerkinElmer liquid scintillation counter at 90% efficiency.

#### Inositol-1-phosphate (IP-1) Formation

HEK-h5HT2A or HEK-h5HT2C cells are used as the tissue source. The day before the experiment, cells are plated in 24-well plates at a density of 400,000 cells per well using DMEM supplemented with charcoal-stripped FetalClone. Drugs are made up in stimulation buffer supplied in the kit. Medium is removed from the well, test compounds or serotonin or antagonist or buffer is added, and the cells are incubated for 1 h. The cells are lysed for 30 min, and 50 μL of cell lysate is added to the IP-1 plates. After the addition of appropriate antibodies, the plates are incubated for 3 h, washed six times, incubated with the substrate for 20 min, and, after termination of the reaction, the plate is read on a plate reader at 450 nm with a correction at 620 nm. Agonists are normalized to the maximal stimulation by serotonin, and antagonists are tested in the presence of 100 nM serotonin and normalized to the inhibition by 10 μM ketanserin (5-HT_2A_R) or 1 μM SB 242084 (5-HT_2C_R).

### Computational Methods

The docking simulations were based on the resolved structure of D_3_R in complex with eticlopride (PDB Id: 3PBL). The protein structure was checked and prepared as reported elsewhere.^40^ The ligands were generated in their protonated state, and their structure was optimized by PM7 semi-empirical calculations as implemented by MOPAC2016 (keywords = PM7 CHARGE = 1.00 PRECISE GEO-OK).^56^ The docking simulations were carried out using PLANTS^57^ focusing the search within a 10 Å radius sphere around the bound eticlopride. For each ligand, 10 poses were generated and evaluated by the ChemPLP scoring function with a speed equal to 1. The same procedure was applied to perform docking simulations on the resolved structure of D_4_R in complex with nemonapride (PDB Id: 5WIV).

For the performed MD runs, the membrane was added to the computed complexes using the CHARMM-GUI server.^58^ The protein was oriented using the PPM server,^59^ and the bilayer was made by phosphatidylcholine (POPC, 70%) and cholesterol (30%). TIP3P water molecules were added to both sides of the membrane, as well as Na^+^ and Cl^–^ ions to reach an ionic concentration of 0.15 M. Amber force fields ff14SB, GAFF, and Lipid17 were used for the proteins, ligands, and lipids, respectively. The systems underwent a three-step minimization: first, the hydrogen atoms were minimized, then the solvent molecules, and finally the whole system, applying restraints (5 kcal/mol·Å) in the α carbons. Then, the systems underwent heating where the temperature was brought to 300 K using the Langevin thermostat, followed by an equilibration phase first using the NPT ensemble with the Berendsen barostat (1 atm) and finally an *NVT* ensemble. The 200 ns production runs were performed with the *NVT* ensemble with a time step of 0.02 fs and the SHAKE algorithm. PME and PBC were applied.^60^

## Supporting Information Available

Cartoon view of the computed complexes for **3** and **9** and rmsd profiles as derived from the performed MD runs for the simulated complexes for **3**, **9**, and **4a** (PDF)

## Abbreviations

DR: dopamine receptor
GPCR: G protein-coupled receptor
PD: Parkinson’s disease
PP: primary pharmacophore
SP: secondary pharmacophore
5-HT_1A_R: 5-HT_1A_ receptor
TLC: thin-layer chromatography
