# Supplementary material for: Scaffold Hybridization Strategy Leads to the Discovery of Dopamine D3 Receptor-Selective or Multitarget Bitopic Ligands Potentially Useful for Central Nervous System Disorders
Source: ACS Chem Neurosci. 2021 Sep 16;12(19):3638–49. doi: 10.1021/acschemneuro.1c00368 (PMC8498988; doi:10.1021/acschemneuro.1c00368)
Supplement: Supplementary file 1 — cn1c00368_si_001.pdf [file cn1c00368_si_001.pdf]

Supporting Information

Scaffold Hybridization Strategy Leads to the Discovery of Dopamine D_3 Receptor-Selective or Multitarget Bitopic Ligands Potentially Useful for Central Nervous System Disorders

Alessandro Bonifazi,[‡,†] Amy H. Newman,[‡] Thomas M. Keck,[‡,⊥] Silvia Gervasoni,[‖] Giulio Vistoli,[‖] Fabio Del Bello,[*,†] Gianfabio Giorgioni,[†] Pegi Pavletić,[†] Wilma Quaglia,[*,†] and Alessandro Piergentili[†]

[‡]*Medicinal Chemistry Section, Molecular Targets and Medications Discovery Branch, National Institute on Drug Abuse – Intramural Research Program, National Institutes of Health, Baltimore, Maryland, 333 Cassell Drive, Baltimore, Maryland 21224*

[⊥] *Department of Chemistry & Biochemistry, Department of Molecular & Cellular Biosciences, Rowan University, 201 Mullica Hill Rd Glassboro, NJ 08028*

[†]*School of Pharmacy, Medicinal Chemistry Unit, University of Camerino, Via S. Agostino 1, 62032 Camerino, Italy*

[‖]*Department of Pharmaceutical Sciences, University of Milan, Via Mangiagalli 25, 20133 Milano, Italy*

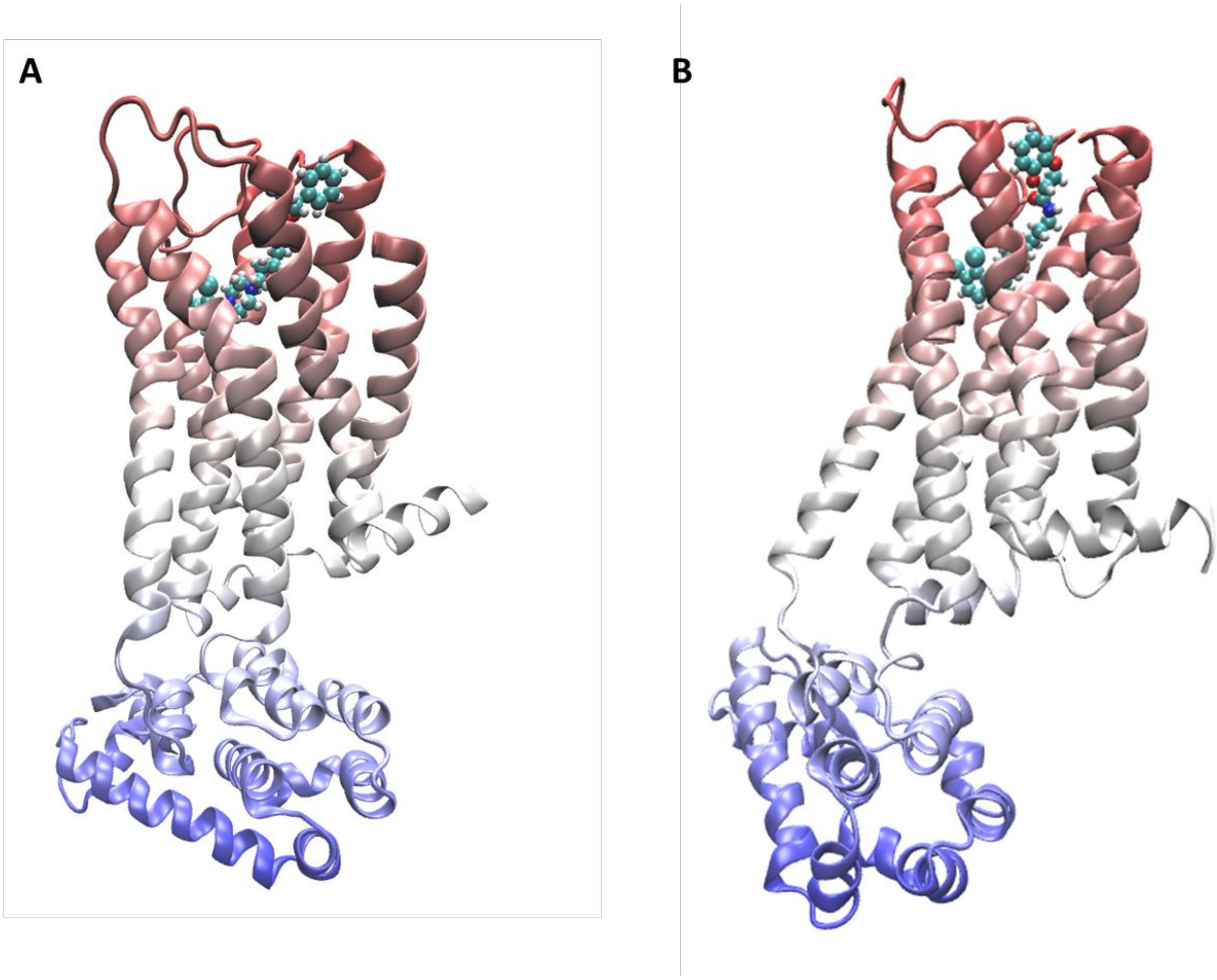

Figure S1. Cartoon view of the computed complexes for **3** (S1A) and **9** (S1B). The backbone is coloured by Y axis (from red for extracellular portion to blue for intracellular domains).

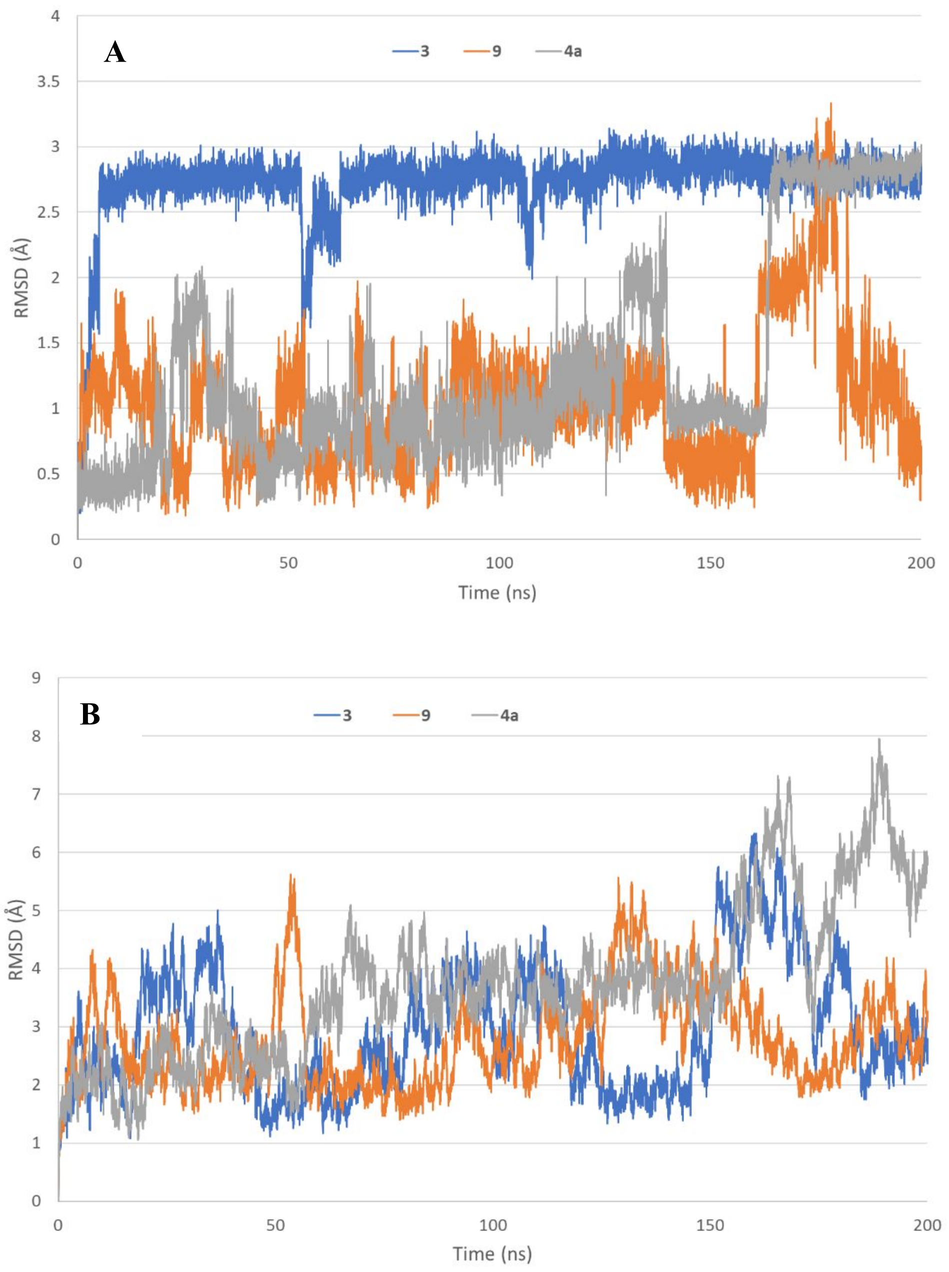

Figure S2: rmsd profiles as derived from the performed MD runs for the three simulated complexes (i.e. **3**, **9** and **4a**) and computed for the ligand's atoms only (S2A) and for the D_3R backbone atoms (S2B)
